# Supplementary material for: Cytotoxic and Genotoxic Effects of Composite Resins on Cultured Human Gingival Fibroblasts
Source: Materials (Basel). 2021 Sep 11;14(18):5225. doi: 10.3390/ma14185225 (PMC8468467; doi:10.3390/ma14185225)
Supplement: Supplementary file 1 [file materials-14-05225-s001.zip › materials-1347396-supplementary.pdf]

materials

MDPI

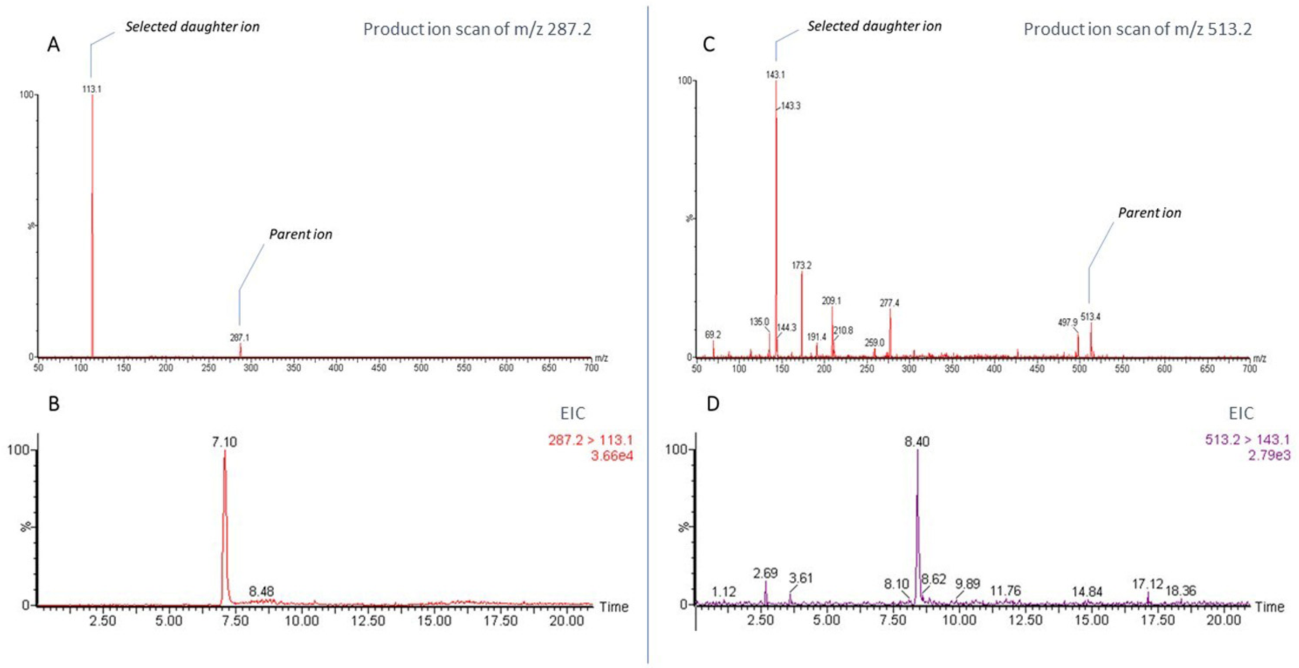

Figure S1. LC-MS/MS data for TEGDMA and Bis-GMA. Panels (**a**) and (**c**) show product ion scans (MS/MS) of m/z 287.2 and m/z 513.2 for TEGDMA and Bis-GMA, respectively; panels (**b**) and (**d**) show the Extracted Ion Chromatogram (EIC) for TEGDMA and Bis-GMA with their retention times (7.10′ for TEGDMA; 8.40′ for Bis-GMA).

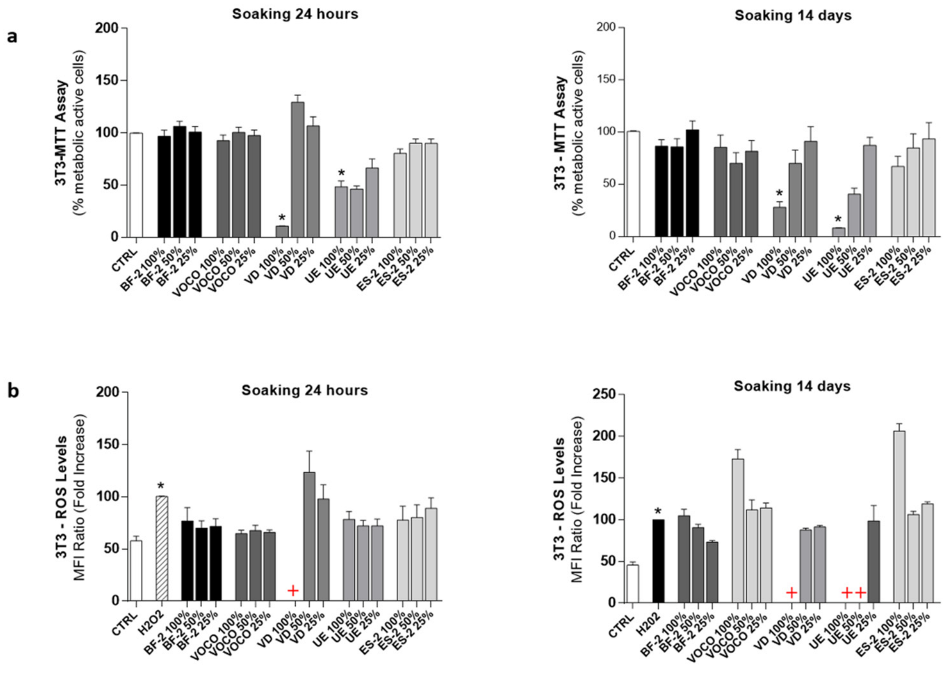

Figure S2. Effects of CRs on Balb/c 3T3 cell line. (**a**) Cells metabolic activity and (**b**) ROS production in Balb/c 3T3 cells following 48 hours of treatment with the 24 hours and 14 days CRs-derived extracts undiluted (100%) or not (50% and 25%). Results, expressed as mean ± error standard (SEM) ($n = 3$). (*$p < 0.05$ *vs* CTRL). The red crosses indicate value not quantifiable (n.q.) in VD and UE groups due to the high level of cells mortality.
